# Spontaneous recovery from sunitinib-induced disruption of sarcomere in human iPSC-cardiomyocytes and possible involvement of the Hippo pathway

**DOI:** 10.1186/s40360-021-00527-5

**Published:** 2021-10-06

**Authors:** Toshikatsu Matsui, Tadahiro Shinozawa

**Affiliations:** Drug Safety Research and Evaluation, Research, Takeda Pharmaceutical Company Limited, 26-1 Muraoka-Higashi 2-chome, Fujisawa, Kanagawa 251-8555 Japan

**Keywords:** Cardiotoxicity, Human induced pluripotent stem cell-derived cardiomyocytes, High-content analysis, Sarcomere disruption, Hippo pathway

## Abstract

**Background:**

Sunitinib is known to cause cardiotoxicity in clinical settings. However, among sunitinib-treated patients experiencing adverse cardiac events, decreased cardiac function was reportedly reversible in > 50% of the patients. We previously showed that anti-cancer drugs such as sunitinib cause marked sarcomere disruption in human induced pluripotent stem cell-derived cardiomyocytes (iPSC-CMs), and the extent of sarcomere disruption can be used to predict drug-induced cardiotoxicity in humans. The aim of this study is to investigate whether the reversibility of sunitinib-induced cardiac events in clinical settings can be mimicked in vitro, and to examine the molecular mechanism responsible for sunitinib-induced cardiotoxicity focusing on the Hippo pathway.

**Methods:**

iPSC-CMs were stimulated with sunitinib for 72 h and the morphology of sarcomere structures were analyzed by high-content analysis before and after sunitinib washout. To examine the involvement of the Hippo pathway in the sunitinib-induced sarcomere disruption, the extent of nuclear localization of YAP1 (yes-associated protein 1, a Hippo signaling target) was determined. iPSC-CMs were also stimulated with sunitinib and a small molecule inhibitor of the Hippo pathway, XMU-MP-1 and sarcomere structures were analyzed.

**Results:**

We observed a spontaneous recovery in cardiac sarcomeres in iPSC-CMs that were significantly disrupted by sunitinib treatment after a 72 h or 144 h washout of sunitinib. The extent of nuclear localization of YAP1 was significantly reduced after sunitinib stimulation and tended to return to normal levels after drug washout. Simultaneous stimulation of iPSC-CM with sunitinib and XMU-MP-1 suppressed the sunitinib-induced disruption of sarcomeres.

**Conclusions:**

These results indicate that iPSC-CMs have the ability to recover from sunitinib-induced sarcomere disruption, and the Hippo pathway plays a role in the process of sunitinib-induced disruption of sarcomere and its recovery. Inhibition of the Hippo pathway may help to develop a co-medication strategy for mitigating the risk of sunitinib-induced adverse cardiac events.

## Background

Sunitinib, a multitargeted kinase inhibitor approved for the treatment of metastatic renal cell carcinoma in humans, is known to be cardiotoxic, resulting in hypertension, electrocardiogram (ECG) changes [1], reduction of left ventricular ejection fraction (LVEF) [2], and heart failure [3] via various mechanisms [4], thereby limiting its clinical use. In contrast, among sunitinib-treated patients experiencing adverse cardiac events, decreased cardiac function was reportedly reversible in > 50% of the patients [5]. Thus, we could develop new strategies to mitigate sunitinib-induced cardiac injuries by recapitulating reversible cardiac injuries and elucidating the molecular mechanisms driving cardiac events in preclinical models. We previously demonstrated that many cancer drugs, including sunitinib, significantly disrupt the sarcomeres in human induced pluripotent stem cell-derived cardiomyocytes (iPSC-CMs), and the extent of disruption could help predict the occurrence of drug-induced cardiotoxicity [6]. Therefore, in the present study, we investigated whether the reversibility of sunitinib-induced cardiac events in clinical settings can be mimicked in vitro by analyzing the sarcomere structure in iPSC-CMs before and after sunitinib washout. In addition, we attempted to identify the molecular pathway involved in sunitinib-induced sarcomere disruption in iPSC-CMs.

The organization of cardiac sarcomeres is strictly regulated by the balance between synthesis, assembly, and sarcomere degradation, to maintain the physiological states of contraction and relaxation [7]. We focused on the Hippo pathway, as it reportedly plays a pivotal role in tissue regeneration, organ size regulation [8], and cardiac regeneration following injury [9]. Though dysregulation of the Hippo pathway is reportedly associated with various cardiac diseases [10], it is unclear whether this pathway is involved in sunitinib-induced adverse cardiac events. Treatment with XMU-MP-1, a small molecule inhibitor of kinases MST1/2 (homologs of Hippo), has been shown to preserve cardiac function following pressure overload in mice [11]. Additionally, XMU-MP-1 conferred tissue repair capability in a liver injury mouse model [12]. Thus, to examine the role of the Hippo pathway in sunitinib-mediated cardiac injury, we focused on the structure of the cardiac sarcomere and nuclear localization of yes-associated protein 1 (YAP1) in iPSC-CMs following stimulation with sunitinib and after washout of the drug. We also analyzed the sarcomere structure after simultaneous simulation with sunitinib and XMU-MP-1 to confirm whether the inhibition of the Hippo pathway has a beneficial effect on sunitinib-induced cardiac injury.

## Methods

### Materials

All reagents and compounds were purchased from either Fujifilm Wako Pure Chemical (Osaka, Japan), Thermo Fisher Scientific (Waltham, MA, USA), or Sigma-Aldrich (St. Louis, MO, USA), unless stated otherwise.

### Cell culture

The human iPSC-derived “iCell Cardiomyocytes^2^” (iPSC-CMs) used in this study and their culture method are the same as those reported in our previous study [6]. Briefly, iPSC-CMs were purchased from Fujifilm Cellular Dynamics International (Tokyo, Japan). The purity of cardiomyocytes was ≥99%. The cells were seeded onto collagen I-coated 96-well plates (Corning, Inc., Corning, NY, USA) at a density of 93,750 cells/cm^2^ using iCell Cardiomyocytes Plating Medium, according to the manufacturer’s protocol. The cells were grown and maintained in 5% CO_2_ at 37 °C. Four hours after seeding, the plating medium was replaced with the manufacturer-provided iCell Cardiomyocytes Maintenance Medium. The maintenance medium was refreshed every two days throughout the experiment.

### Compound preparation and stimulation

After the iPSC-CMs were cultured for 5–6 d, they were stimulated with sunitinib with or without XMU-MP-1 for 72 h. Both sunitinib and XMU-MP-1 were dissolved in 100% (v/v) dimethyl sulfoxide (DMSO). The compounds were added to the wells of a 96-well plate to obtain the final concentrations 0.3 μM, 1, μM, or 3 μM for either sunitinib or XMU-MP-1. The final DMSO concentration was 0.1% (v/v). The cells were then cultured with each compound for 72 h. To evaluate the effect of drug washout on sarcomere structure, the sunitinib-containing medium was removed and replaced with fresh drug-free medium after 72 h of sunitinib stimulation.

### Paraformaldehyde (PFA) fixation and immunostaining

The PFA fixation and immunostaining processes were performed according to our previously reported methods [6]. After drug stimulation and washout, cells were fixed with 2% PFA for 15 min at 37 °C then washed twice with PBS. After blocking the cells with blocking buffer [1× PBS Tween-20 (28,352; Thermo Fisher Scientific) containing 1.5% (v/v) bovine serum albumin (A9205; Sigma-Aldrich)] for 30 min at room temperature (22–25 °C), the cells were incubated with a polyclonal antibody against rabbit alpha-actinin (AB90776; Abcam, Cambridge, UK), diluted with blocking buffer to a final concentration of 2 μg/mL, at 4 °C for 18 h. To analyze YAP1 nuclear localization, we used a rabbit monoclonal antibody against active YAP1 (AB205270; Abcam), diluted with blocking buffer to a final concentration of 0.2 μg/mL, at 4 °C for 18 h. After discarding the primary antibody solution, cells were washed twice with PBS and incubated with a secondary antibody solution, including goat anti-rabbit IgG (H + L) secondary antibody (A11034, Alexa Fluor 488, 1:400) and Hoechst 33342 (1:800; Thermo Fisher) at 4 °C for 1 h. Finally, cells were washed twice with PBS, and each well was filled with PBS before performing high-content analysis (HCA).

### Assessment of cardiac sarcomere structure and YAP1 nuclear localization using HCA

The protocol for the analysis of the cardiac sarcomere structure has been described previously [6]. Briefly, automated fixed-cell fluorescent images of immunostained cells were obtained using the IN Cell Analyzer 6500 device (GE Healthcare Japan, Tokyo, Japan) using a 60× or 4× objective lens. To analyze the sarcomere structure, nine image fields per well were acquired using the 60× objective lens. To analyze YAP1 nuclear localization, six image fields per well were acquired using the 4× objective lens. For all image analyses, we used the IN Cell Developer Tool Box software (GE Healthcare). The number of cell nuclei per image field was calculated by observing the pattern of Hoechst-stained nuclei, and the total number of cell nuclei per well were determined. For quantification of the sarcomere morphology, we measured the lengths of all the Z lines of the sarcomeres observed in each cell by analyzing the image exhibiting the immunofluorescence signal of alpha-actinin, then we calculated the average length of all recognized Z lines per cell. Next, to determine whether the observed cells were healthy or morphologically damaged, based on the average length of all recognized Z lines per cell, we counted the number of cells per field that differed concomitantly according to changes in the cutoff value, to determine the average length of Z lines of cells. By searching for an optimal cutoff value, we defined cells with an average Z line sarcomere length > 3.1 μm as cells with a “ well-organized sarcomere.” Then, we counted the number of cells with well-organized sarcomeres per well using the analysis software, and determined the ratio of the number of cells with well-organized sarcomeres to the total number of cells observed in each well, which we defined as the “Healthy sarcomere index (HSI)” in all experiments. To determine the HSI, approximately 200 cells were analyzed in each well (because of high magnification (× 60)), one well was used for each data point, and five or six wells were used as the data for one group (*n* = 5–6 wells/group) Similarly, the ratio of HSI in the treatment group to that in the control group was calculated. For quantification of the YAP1 nuclear localization, immunofluorescence intensity of YAP1 in the cytoplasm and nuclei, respectively, were measured. Then, cells having a significantly more intense signal of YAP1 in the nuclei than in the cytoplasm were defined as cells showing nuclear localization of YAP1. Then, the degree of nuclear localization of YAP1 was determined by calculating the ratio of the number of cells showing nuclear localization of YAP1 to the total number of cells observed in each well and expressed as the relative ratio to the control group. For the quantitation of nuclear localization of YAP1, approximately 4000 cells were analyzed in each well (because of low magnification (× 4)), one well was used for each data point, and five or six wells were used as the data for one group (*n* = 5–6 wells/group). Since the iPSC-CMs from Fujifilm Cellular Dynamics International have been previously reported to be predominantly mononuclear cells [13], we conducted image analyses in all the experiments assuming that the iPSC-CMs are mononuclear cells.

### Statistical analysis

All results are presented as mean ± SD values and all graphs were created using the GraphPad Prism 8 software. Statistical analyses were performed using EXSUS2014 software. The HSI and degree of YAP1 nuclear localization between the DMSO and the drug-treated group were compared using Williams’ test or Shirley-Williams’ test, depending on whether the variances were heterogeneous or not. Statistical significance was set at *p* < 0.025. Student’s t-test or the Wilcoxon rank sum test was conducted to perform comparisons between DMSO and 3 μM sunitinib-treated groups in Fig. 3D; *p* < 0.05 was considered statistically significant.

## Results

**Fig. 1:**
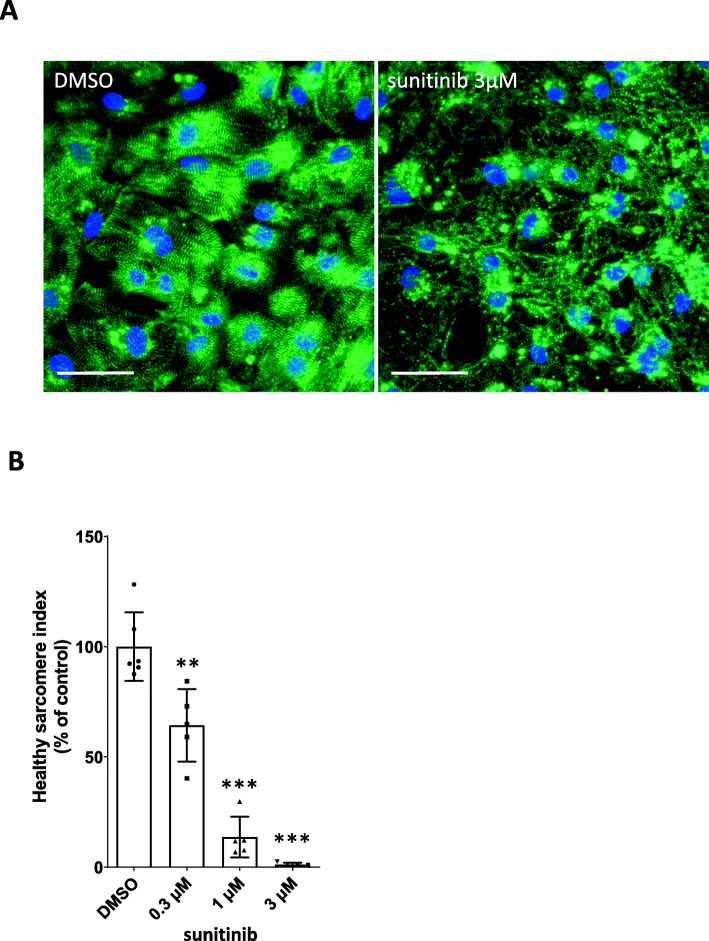
Sunitinib-induced disruption of cardiac sarcomeres in iPSC-CMs (**A**) Typical immunostaining results of iPSC-CMs following exposure to DMSO (control) or 3 μM sunitinib for 72 h. Nuclei are shown in blue, while sarcomeres are shown in green (60×; scale bar: 50 μm). (**B**) Quantitative analysis of sarcomere morphology via high-content image analysis. Data represent the percent change in the HSI of iPSC-CMs relative to the control and are expressed as mean ratios ± SD. Approximately 200 cells were analyzed in each well, one well was used for each data point, and five or six wells were used as the data for one group (*n* = 5–6 wells/group). **: *p* < 0.005, ***: *p* < 0.0005 versus control group using Shirley-Williams’ multiple comparison test

Regular striped staining images of sarcomeric Z lines exhibiting the immunofluorescence signal of alpha-actinin were seen in the DMSO-treated control groups, whereas dot-like staining images showing apparent disruption of sarcomeric Z lines were observed in the sunitinib-treated group (Fig. 1A). Sunitinib treatment decreased the percentage of cells with well-organized sarcomeres in a concentration-dependent manner, as indicated by the reduction in HSI (Fig. 1B). Seventy-two hours after stimulation with sunitinib, the HSI values of groups treated with 0.3 μM, 1 μM, and 3 μM sunitinib were 64.3, 13.7, and 1.1% of the control group, respectively. This result suggests that sunitinib caused sarcomere disruption in iPSC-CMs.

**Fig. 2:**
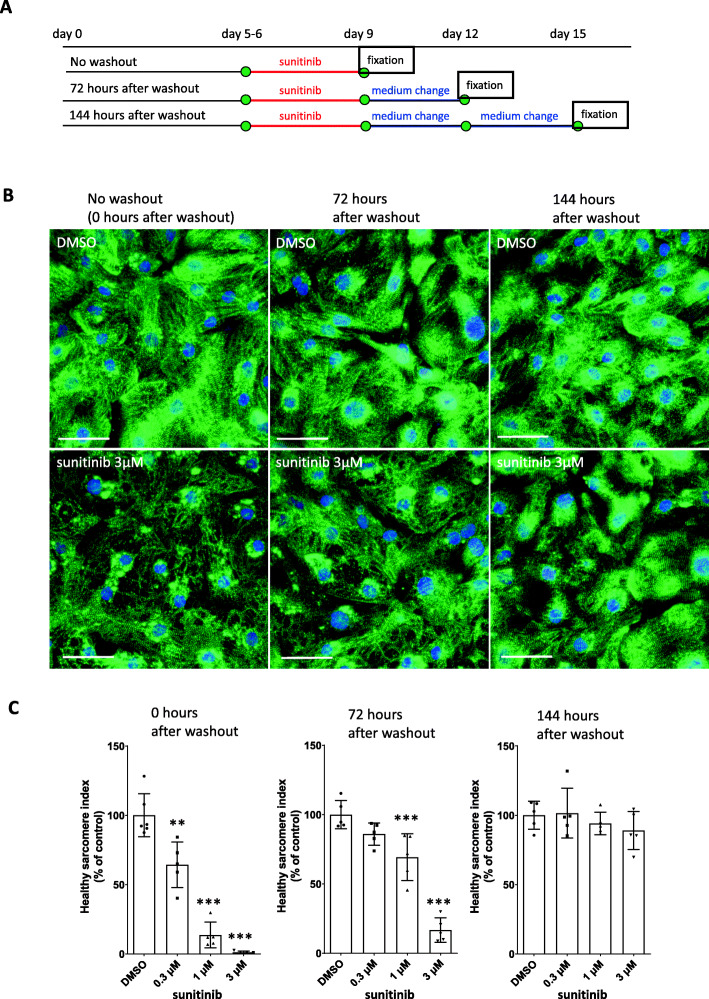
Spontaneous recovery from sunitinib-induced disruption of cardiac sarcomeres (**A**) Experimental design for analysis of sarcomere morphology following sunitinib washout. (**B**) Representative immunostaining results for iPSC-CMs following exposure to DMSO or sunitinib (leftmost image) for 72 h; images were obtained 72 h (middle image) and 144 h (rightmost image) after DMSO or sunitinib washout (60×; scale bar: 50 μm). (**C**) Quantitative analysis of sarcomere morphology via high content image analysis. Data represent the percent change in the HSI of iPSC-CMs relative to the control and are expressed as the mean ratios ± SD. Approximately 200 cells were analyzed in each well, one well was used for each data point, and five or six wells were used as the data for one group (*n* = 5–6 wells/group). **: *p* < 0.005, ***: *p* < 0.0005 versus control group using Shirley-Williams’ multiple comparison test (0 h after washout). ***: *p* < 0.0005 versus control group using Williams’ multiple comparison test (72 h after washout)

Next, we analyzed whether iPSC-CMs had the ability to spontaneously recover from sunitinib-induced sarcomere disruption by analyzing sarcomere morphology following stimulation with sunitinib and subsequent drug washout. We set two different time points (72 h and 144 h) for sarcomere analysis after sunitinib washout (Fig. 2A). Immediately after 72 h of sunitinib stimulation, sarcomeric Z lines were observed as dot-like staining images indicating sarcomere disruption in most iPSC-CMs, but after 72 h of drug washout, the dot-like staining images partially changed to stripe-like staining images, and after 144 h of washout, the dot-like staining images almost disappeared and almost completely changed to normal stripe-like staining images (Fig. 2B). The HSI values of groups treated with 0.3 μM, 1 μM, and 3 μM sunitinib 72 h after drug washout were 86.0, 69.3, and 16.7% of the control group, respectively. Further, 144 h after drug washout, the HSI values of groups treated with 0.3 μM, 1 μM, and 3 μM sunitinib were 101.5, 94.1, and 89.1% of the control group, respectively, and there was no statistical significance in the HSI between treatment groups (Fig. 2C).

**Fig. 3:**
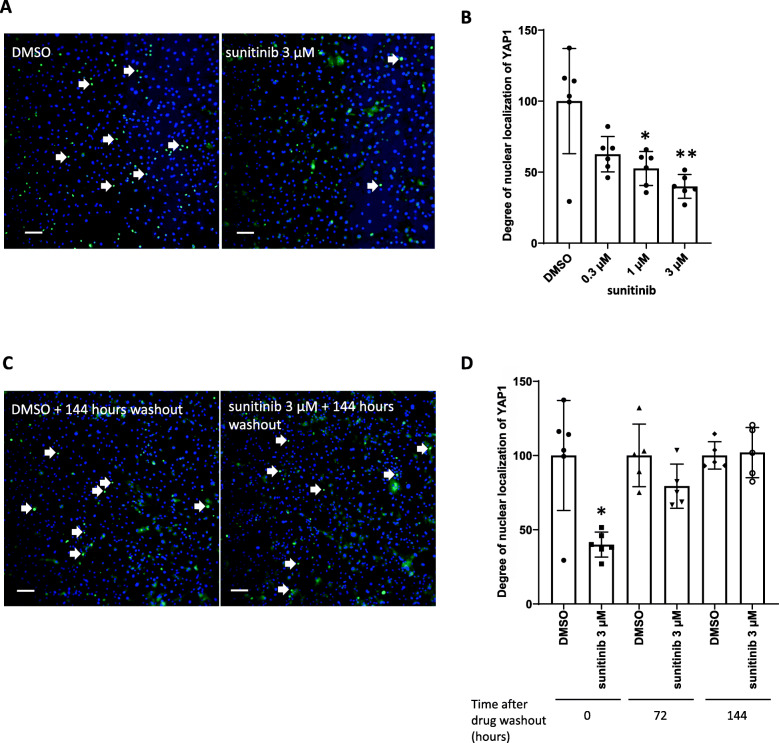
Nuclear localization of YAP1 in iPSC-CMs 72 h after stimulation with sunitinib and 72 h or 144 h after drug washout (**A**) Typical immunostaining results for iPSC-CMs following exposure to DMSO (control) or 3 μM sunitinib for 72 h. Nuclei are shown in blue, and nuclear localization of YAP1 is shown in green (white arrows) (4×; scale bar: 100 μm). (**B**) Quantitative analysis of the extent of nuclear localization of YAP1 via high-content image analysis. Data represent the percent change in the degree of nuclear localization of YAP1 in iPSC-CMs relative to the control and are expressed as mean ratios ± SD. Approximately 4000 cells were analyzed in each well, one well was used for each data point, and six wells were used as the data for one group (*n* = 6 wells/group). *: *p* < 0.025, **: *p* < 0.005 versus control groups using Shirley-Williams’ multiple comparison test. (**C**) Representative immunostaining results for iPSC-CMs obtained 144 h after washout of 3 μM sunitinib (4×; scale bar: 100 μm). (**D**) Quantitative analysis of the degree of nuclear localization of YAP1 72 h or 144 h after washout of 3 μM sunitinib via high-content image analysis. Data are mean ratios ± SD. Approximately 4000 cells were analyzed in each well, one well was used for each data point, and five or six wells were used as the data for one group (*n* = 5–6 wells/group). *: *p* < 0.05 versus control group using Wilcoxon rank sum test

We next investigated the molecular pathways involved in the disruption or recovery of sarcomere structure following sunitinib-mediated injury in iPSC-CMs. We focused on the Hippo pathway and examined whether an alteration in one of the molecular components of the pathway was involved in sunitinib-induced sarcomere disruption and/or spontaneous recovery. Seventy-two hours after stimulation with 3 μM sunitinib, a reduction in the degree of YAP1 nuclear localization was clearly observed (Fig. 3A). The extent of YAP1 nuclear localization in iPSC-CMs following stimulation with 0.3 μM, 1 μM, or 3 μM sunitinib was 62.6, 52.6, and 40.0% of the control group, respectively (Fig. 3B). Then, we investigated the extent of changes in YAP1 nuclear localization following sunitinib washout. At 144 h after washout of 3 μM sunitinib, no apparent difference was observed in the extent of YAP1 nuclear localization between control and sunitinib treatment groups (Fig. 3C). The degree of YAP1 nuclear localization 72 h and 144 h after 3 μM sunitinib washout was 79.3 and 102.0% of the control, respectively (Fig. 3D).

**Fig. 4:**
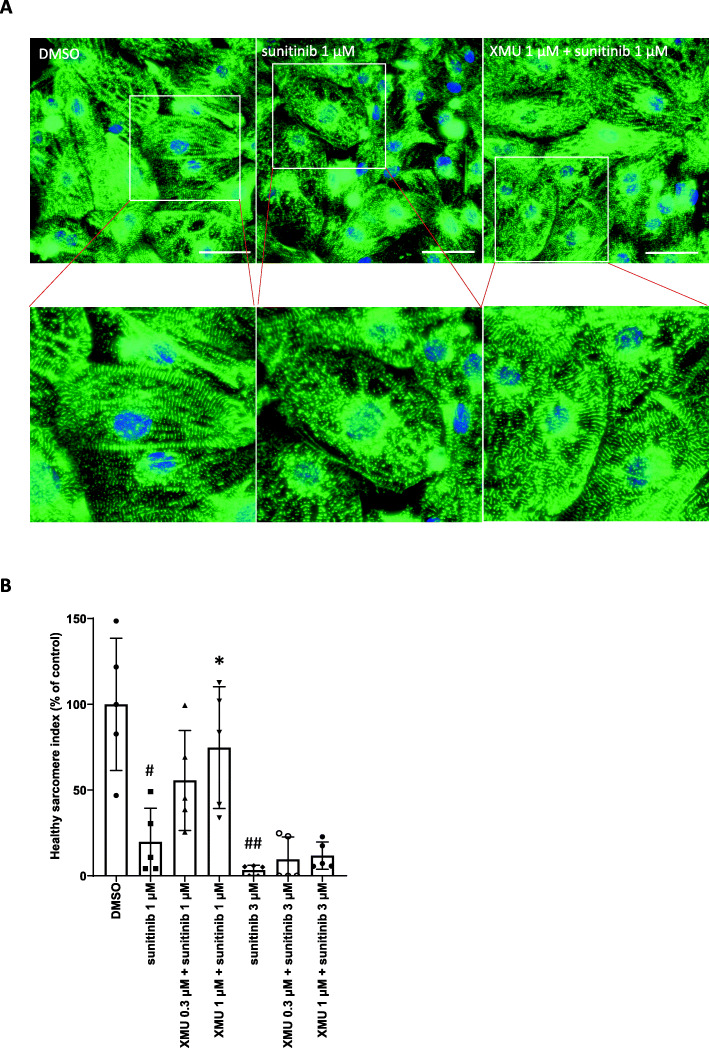
Mitigation of sunitinib-induced sarcomere disruption via the inhibition of MST1/2 using XMU-MP-1 (**A**) Representative immunostaining results for iPSC-CMs following exposure to DMSO or sunitinib with or without XMU-MP-1 for 72 h. The leftmost, middle, and rightmost images represent the DMSO, 1 μM sunitinib, and sunitinib/XMU-MP-1 co-treated groups, respectively (60×; scale bar: 50 μm). (**B**) Quantitative analysis of sarcomere morphology via high-content image analysis. Data represent the percent change in the HSI of iPSC-CMs relative to the control and are expressed as the mean ratios ± SD. Approximately 200 cells were analyzed in each well, one well was used for each data point, and five wells were used as the data for one group (*n* = 5 wells/group). #: *p* < 0.025, ##: *p* < 0.005 versus control groups using Shirley-Williams’ multiple comparison test. *: *p* < 0.025 versus 1 μM sunitinib group using Williams’ multiple comparison test

From these results, we assumed that the Hippo pathway may be involved in the sunitinib-induced sarcomere disruption, and spontaneous recovery, observed after drug washout. To further examine the involvement of the Hippo pathway in the disruption and/or recovery process, we modulated the pathway with a small molecule kinase inhibitor, XMU-MP-1, with the expectation that this could recapitulate spontaneous recovery of cardiac sarcomeres. We added either 1 μM or 3 μM sunitinib with or without 0.3 μM or 1 μM XMU-MP-1 for 72 h. XMU-MP-1 suppressed sunitinib-induced sarcomere disruption in a concentration-dependent manner (Fig. 4A and B). Compared to the control (DMSO) group, the HSI values of groups treated with 1 μM sunitinib, 1 μM sunitinib with 0.3 μM XMU-MP-1, and 1 μM sunitinib with 1 μM XMU-MP-1 were 19.8, 55.7, and 74.7%, respectively. On the other hand, the HSI of the group treated with 3 μM sunitinib alone was 3.3% compared to the control group. For the groups treated with 3 μM sunitinib and either 0.3 μM or 1 μM XMU-MP-1, the HSI values were 9.6 and 11.8%, respectively. Stimulation with ≥3 μM XMU-MP-1 resulted in a decreasing trend in the number of cell nuclei (data not shown).

## Discussion

Currently, there are almost no radical treatments available for cancer drug-induced cardiotoxicity, and the demand for prevention of chemotherapy-induced cardiotoxicity is growing [14]. The molecular mechanisms responsible for sunitinib-induced cardiotoxicity are complicated and, despite their severity, remain largely unclarified. In this study, we focused on the morphology of cardiac sarcomeres following stimulation with sunitinib, since cardiac sarcomeres are major components of myofibrillar fibers and play a pivotal role in myocardial contraction and relaxation [15]. We, for the first time, discovered that a recovery of the sunitinib-induced disruption in cardiac sarcomeres to an almost normal state in iPSC-CMs can be achieved, following drug washout. This suggests that our in vitro assay can mimic the reversibility of LVEF decreases observed in sunitinib-treated patients [5].

At present, limited information is available regarding the reversibility of sunitinib-induced cardiac injury in clinical settings. A previous study showed that, in cancer patients treated with sunitinib and exhibiting cardiac adverse events (e.g., reduction of ejection fraction, ECG changes), recovery was observed after the interruption of sunitinib treatment and administration of cardiovascular treatments including medical intervention, coronary angiography, and surgical procedures [1]. However, because it is unknown whether this recovery was achieved through the interruption of sunitinib treatment or cardiovascular management, it is difficult to accurately understand the potential and extent of spontaneous recovery from cardiac events in sunitinib-treated patients. Thus, to further demonstrate the relevance of these findings in iPSC-CMs to the clinical setting, and to gain a better understanding of the mechanism behind the recovery process, an assessment of drug-induced functional readouts (e.g., contractility, beat rate, extra field potential) in iPSC-CMs would be required in future studies.

The Hippo pathway negatively regulates the activity of transcriptional co-activator YAP1 [16], which controls the transcription of genes related to cell proliferation, apoptosis, and cell fate [17]. We observed a significant reduction in the extent of nuclear localization of YAP1 following sunitinib stimulation. Sunitinib inhibits various receptor protein-tyrosine kinases, including VEGF and PDGF [18], which are both known to regulate the Hippo pathway [17]. Indeed, the pharmacological inhibition of PDGF causes the redistribution of YAP1 from the nucleus to the cytosol and downregulates YAP1 target genes [19]. Therefore, one of the causes of the reduction in YAP1 nuclear localization following stimulation with sunitinib may be due to the inhibition of VEGF and/or PDGF. Further studies using various VEGF or PDGF inhibitors would clarify the relationship between the degree of reduction in nuclear localization of YAP1 and the extent of sarcomere injury. Interestingly, the level of YAP1 nuclear localization, which had been reduced after treatment with 3 μM sunitinib, became almost normal 144 h after drug washout. Since this washout period resulted in the complete recovery of disrupted sarcomeres, the increased nuclear localization of YAP1 may be related to the recovery process of injured sarcomeres in iPSC-CMs.

XMU-MP-1 is a potent small-molecule kinase inhibitor of MST1/2 [12]. We found that XMU-MP-1-mediated inhibition of the Hippo pathway only slightly mitigated the disruption of cardiac sarcomere caused by 3 μM sunitinib exposure, as opposed to the sarcomere disruption caused by 1 μM sunitinib. Therefore, in addition to the Hippo pathway, other molecular pathways may also be involved in the spontaneous recovery of cardiac sarcomeres. Indeed, as described earlier, various pathways or molecules, such as autophagy-related pathways [20], proteases [21], and ubiquitin-proteasome pathways [22] are known to be involved in the degradation, synthesis, and assembly of the cardiac sarcomere [7]. Although their functional significance in the disease setting is still unknown, the modulation of each pathway using specific inhibitors may also affect the sarcomere structure, even after stimulation with sunitinib. Furthermore, global gene or protein expression analysis would allow us to further decipher the molecular pathways responsible for the recovery of cardiac sarcomeres after sunitinib-induced injury.

Taken together, we observed that recovery from disruption of the cardiac sarcomere and reduction of nuclear localization of YAP1 caused by sunitinib, could occur following drug washout. Importantly, a small molecule inhibitor of the Hippo pathway, XMU-MP-1, suppressed the sunitinib-induced disruption of cardiac sarcomeres. These findings help understand the therapeutic potential of inhibition of the Hippo pathway in sunitinib-induced cardiac injury. Furthermore, the clarification of the molecular mechanism underlying the recovery of cardiac sarcomeres from a sunitinib-induced disruption would enable discover and development of novel drug targets for the treatment of sunitinib-drug-induced cardiotoxicity.

## Conclusions

**Fig. 5:**
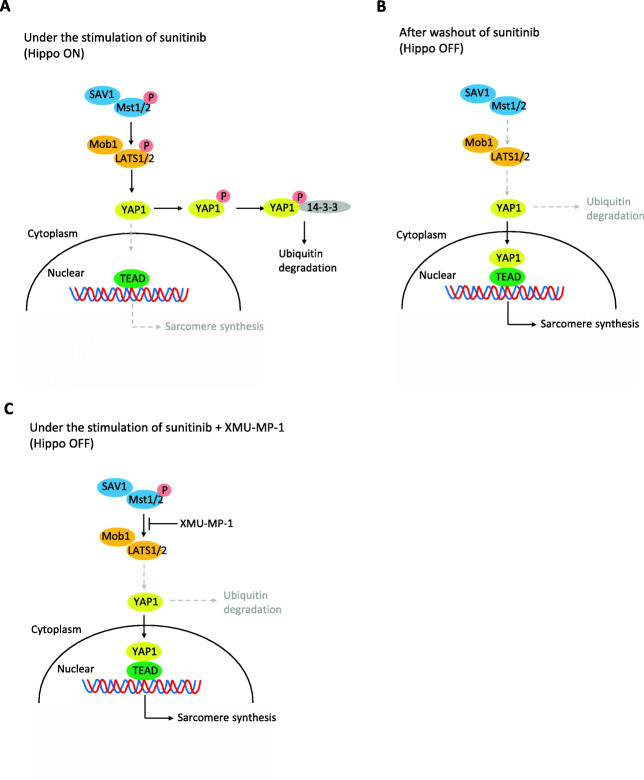
Schematic illustration of the proposed mechanism behind mitigation of sunitinib-induced disruption of sarcomere by inhibition of the Hippo pathway (**A**) Stimulation with sunitinib causes reduction of nuclear localization of YAP1 as well as disruption of sarcomere in iPSC-CM probably through activation of the Hippo pathway. (**B**) Washout of sunitinib normalizes the activated status of the Hippo pathway and the extent of nuclear localization of YAP1 as well as sarcomere disruption returns to normal level possibly as a result of sarcomere synthesis. (**C**) XMU-MP-1 is considered to mimic the condition of sunitinib washout as it is an inhibitor of kinase MST1/2. Indeed, XMU-MP-1 suppressed the sunitinib-induced disruption of sarcomere

The present findings demonstrate that iPSC-CMs have the ability to recover from sunitinib-induced sarcomere disruption, suggesting that our in vitro assay can mimic the reversibility of LVEF decreases observed in sunitinib-treated patients. This study also indicates that the Hippo pathway is involved in the process of sunitinib-induced disruption of sarcomere and its recovery after drug washout (Fig. 5 A, B and C). Additionally, inhibition of the Hippo pathway with XMU-MP-1 suppressed the sunitinib-induced disruption of sarcomere. These results suggest that inhibition of the Hippo pathway may lead to the development of a co-medication strategy to reduce the risk of sunitinib-induced adverse cardiac events.

## Data Availability

The datasets used and/or analyzed during the current study are available from the corresponding author upon reasonable request.

## Abbreviations

iPSC-CMs: induced pluripotent stem cell-derived cardiomyocytes
YAP: yes-associated protein
HSI: healthy sarcomere index
